# Potential role of cervicovaginal extracellular particles in diagnosis of endometriosis

**DOI:** 10.1186/s12917-015-0513-7

**Published:** 2015-08-08

**Authors:** Dillon C. Muth, Melissa A. McAlexander, Lauren J. Ostrenga, Nathan M. Pate, Jessica M. Izzi, Robert J. Adams, Kelly A. Metcalf Pate, Sarah E. Beck, Baktiar O. Karim, Kenneth W. Witwer

**Affiliations:** Department of Molecular and Comparative Pathobiology, The Johns Hopkins University School of Medicine, Baltimore, Maryland USA

## Abstract

**Background:**

Macaques are an excellent model for many human diseases, including reproductive diseases such as endometriosis. A long-recognized need for early biomarkers of endometriosis has not yet resulted in consensus. While biomarker studies have examined many bodily fluids and targets, cervicovaginal secretions have been relatively under-investigated. Extracellular vesicles (EVs, including exosomes and microvesicles) are found in every biofluid examined, carry cargo including proteins and RNA, and may participate in intercellular signaling. Little is known about EVs in the cervicovaginal compartment, including the effects of reproductive tract disease on quantity and quality of EVs.

**Case presentation:**

In September 2014, a 9-year-old rhesus macaque was diagnosed with endometriosis at The Johns Hopkins University School of Medicine. Ultrasound-guided fine needle aspiration of a cyst and subsequent laparotomy confirmed diagnosis. The animal was sent to necropsy following euthanasia for humane reasons. Perimortem vaginal swabs and cervicovaginal lavages were obtained. Using a combination of methods, including ultracentrifugation and NanoSight visualization technology, approximate numbers of EVs from each sample were calculated and compared to populations of EVs from other, reproductively normal macaques. Fewer EVs were recovered from the endometriosis samples as compared with those from reproductively healthy individuals.

**Conclusion:**

To our knowledge, this is the first examination of EVs in primate cervicovaginal secretions, including those of a macaque with endometriosis. This case study suggests that additional research is justified to determine whether quantification of EVs—or their molecular cargo—in cervicovaginal lavage and vaginal swabs may provide a novel, relatively non-invasive diagnostic for primate endometrial disease or other reproductive tract diseases.

## Background

Endometriosis is a benign but debilitating disease of humans and other primates that affects the reproductive tract and has a reported incidence rate of up to 10 % in females of reproductive age [1]. Generally, the term endometriosis is used to describe the condition in which endometrial cells (glands or stroma) develop ectopically, or outside of the uterine cavity [2]. Symptoms of endometriosis include chromic abdominal and pelvic pain, dysmenorrhea, as well as subfertility and likely eventual infertility [3, 4]. Treatment protocols have not been standardized, but typically include surgical removal of ectopic tissue as well as removal of associated adhesions.

The current gold standard for diagnosis of the disease combines laparoscopic evaluation and biopsy. In humans, endometriosis is thought to be prevalent for an average 8 to 11 years before diagnosis [5] and multiple years after the appearance of symptoms [6, 7]. Earlier detection would likely make surgical treatment more effective, afford individuals affected by the disease more options for treatment, and assist in the search for factors involved in pathogenesis. Indeed, the urgent need for early diagnosis was recognized well over a half-century ago [8].

Despite a burgeoning literature on the topic, as yet there is no consensus on early, non-invasive diagnosis methods. Diagnostic markers have been sought in peritoneal, follicular, and endometrial fluid, in urine, and in blood cells and cell-free fractions (see reviews [6, 7, 9–12]). Employment of ultrasound, MRI [13, 14] and measurement of electrical resistance of dermal-visceral zones [15] have also been studied. Recently, attention has turned to circulating miRNAs, which have been reported as biomarkers of various diseases [16]. At least three published studies [17–19] have found an association between endometriosis and differential regulation of specific miRNAs in circulation, although caution in interpretation has been urged due to estrogen sensitivity of some miRNAs [20]. Remarkably, there is no overlap of miRNA biomarkers reported in the different studies. Indeed, one of the studies reported differential expression of miR-16, which was elsewhere used as an invariant control. The lack of concordance between circulating miRNA biomarker studies has previously been reported for conditions including breast cancer [21, 22], and may be due to the high non-specific background of miRNAs that are not associated with disease or are associated with many conditions [23]. Since miRNAs are protected in biofluids by various molecular carriers [24, 25], it may be possible to achieve greater discrimination by focusing on extracellular particles, such as extracellular vesicles (EVs) that may be specifically released from cells [22].

EVs are lipid bilayer vesicles [26] that carry a varied cargo including nucleic acids, proteins, metabolites, and lipids. Release of EVs is thought to have a variety of functions, including disposal of harmful cellular contents and transmission of regulatory molecules in a form of intercellular communication. While determining levels of specific EV cargoes such as miRNAs may be useful in diagnosis, an initial study might yield results with even simpler measurements such as EV particle or EV protein concentration. Indeed, such measurements have previously achieved reported success in ovarian cancer [27].

We hypothesized that a promising potential avenue for achieving early diagnosis of reproductive tract diseases including endometriosis might involve examination of total extracellular particles and EVs in cervicovaginal secretions. In this report, we examined particle concentration in total extracellular particle and EV-enriched ultracentrifuged fractions from cervicovaginal swabs (CVS) and cervicovaginal lavage (CVL) from an individual with endometriosis. We compared these data with results obtained from reproductively normal nonhuman primates, observing apparent disease-associated differences in particle concentrations. To our knowledge, this is the first report of extracellular particle and EV-enriched particle fractions in cervicovaginal secretions from any species.

## Case presentation

**Fig. 1:**
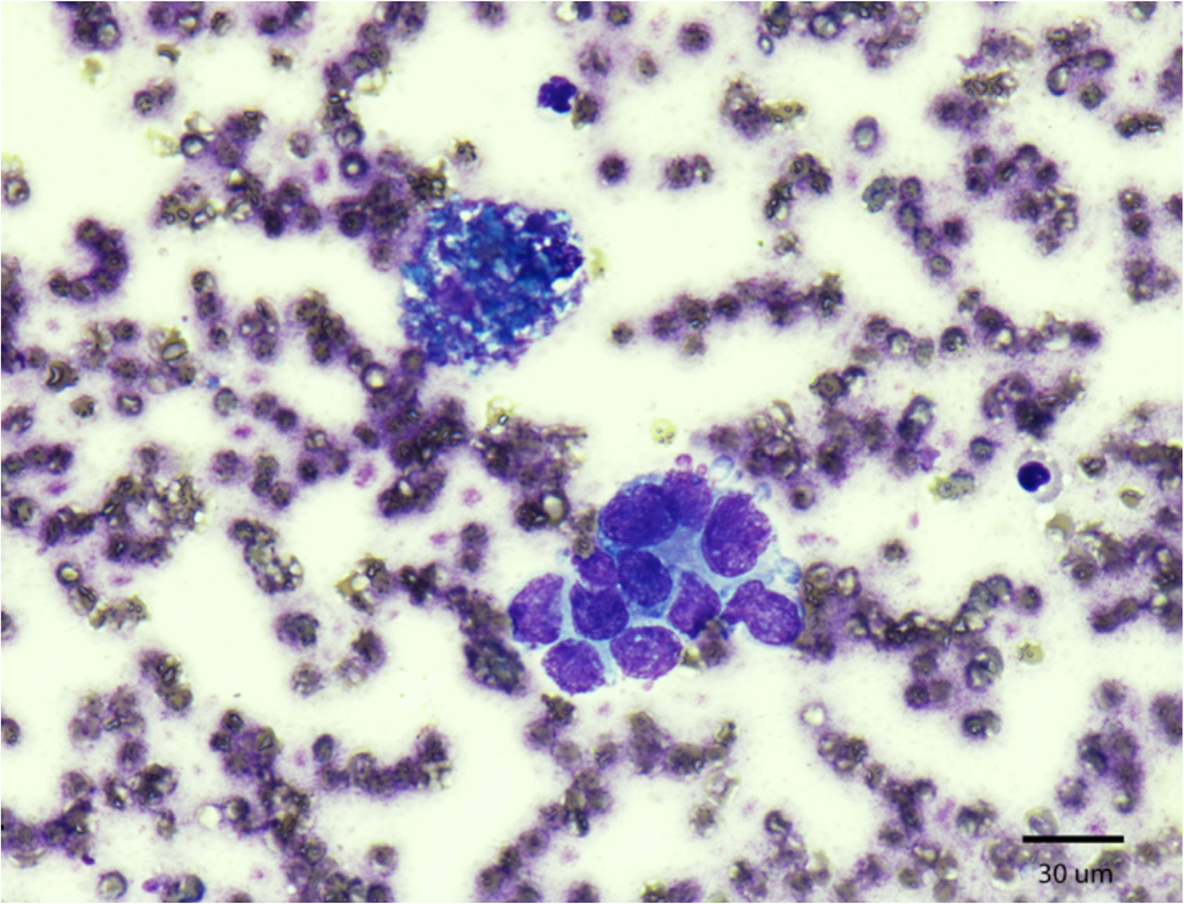
Cytology of fine needle aspirate obtained from abdominal mass. Characteristic hemosiderin-laden macrophages are noted along with a tight cluster of cells with dense, large nuclei and minimal cytoplasm, consistent with endometrial-like cells on a moderate background of erythrocytes. Diff-Quick stain (40X)

A 9-year-old female rhesus macaque (*Macaca mulatta)* with a history of a previous caesarian section presented with a large, firm uterine mass on abdominal and rectal palpation at routine semi-annual physical exam. All animal studies were approved by the Johns Hopkins University Institutional Animal Care and Use Committee and conducted in accordance with the Weatherall Report, the Guide for the Care and Use of Laboratory Animals, and the USDA Animal Welfare Act. Abdominal ultrasound revealed numerous cysts near the uterus. Fine needle aspirate of one of the cysts revealed dark red-brown fluid that on cytology contained numerous degenerate red blood cells, foamy macrophages with hemosiderin, scattered neutrophils and lymphocytes as well as numerous clusters of endometrial-like cells (chocolate cyst) (Fig. 1). A laparotomy was subsequently performed, which revealed extensive cyst formation localized to the ovaries; numerous adhesions extending from and between the reproductive tract and the bladder and omentum were observed. Euthanasia was elected due to poor prognosis.

### Pathologic findings

**Fig. 2:**
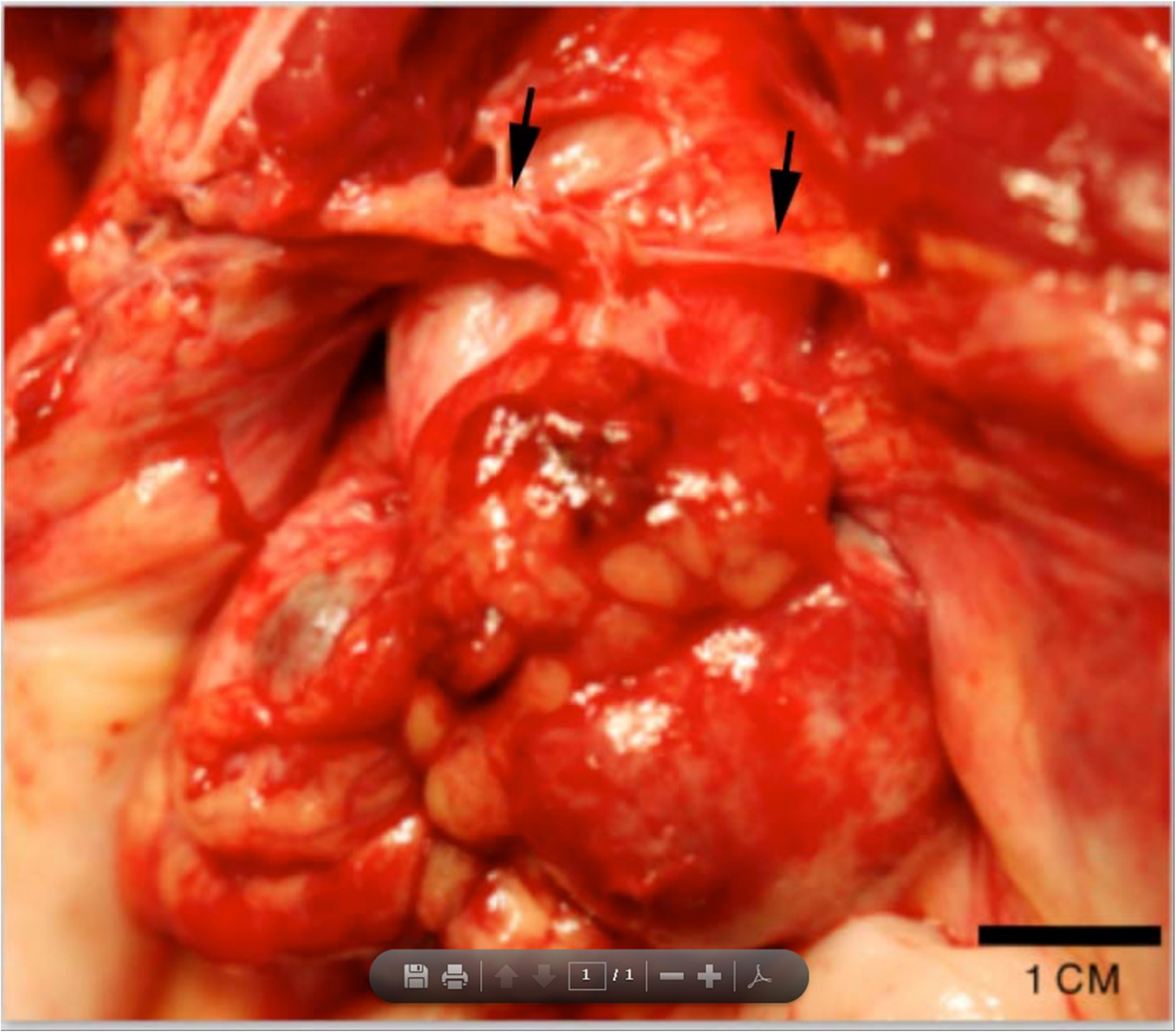
Abdominal Mass. Multiple, large cystic and blood-filled nodules were present in the abdominal cavity along with numerous fibrous adhesions (arrows)

On necropsy, the animal was in good postmortem condition and good body condition (BCS 3/5). The uterus was markedly thickened and nodular with a prominent “chocolate brown” cystic structures on the serosal ventral surface. Dissection between the uterus body, uterine horns, and ovaries was not possible due to the extensive adhesions (Fig. 2). One ovary was grossly discernable and exhibited multiple simple cysts as well as a cyst containing “chocolate brown” material.

**Fig. 3:**
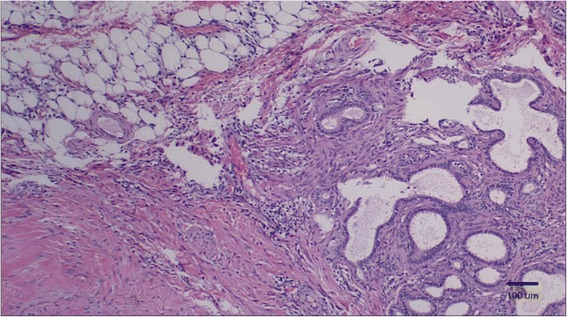
Abdominal Wall. Numerous poorly organized and ectacic endometrial glands were haphazardly arranged within dense connective tissue. H&E stain (10X)

Ovarian histology confirmed initial examination impressions of endometriosis: Transmurally disrupting and expanding the parenchyma of the bladder and uterus and also impacting the mesentery of the retroperitoneal space were multiple, unencapsulated, infiltrative islands of ectopic endometrial glands surrounded by densely cellular endometrial stromal tissue. The endometrial glands were lined by simple columnar epithelial cells with a moderate amount of clear to pale eosinophilic cytoplasm. The endometrial stroma was composed of spindle cells with indistinct cell borders, scant eosinophilic cytoplasm and an oval nucleus with finely stippled chromatin. Occasionally glands contained moderate numbers of macrophages, neutrophils, erythrocytes, and cellular debris. Within the retroperitoneal mesenteric adipose tissue, multifocal areas of hemorrhage, degenerate neutrophils, and necrotic debris is appreciated along with plump mature reactive fibroblasts (adhesions). The adjacent lymph node contained increased number of histiocytes, which often demonstrated erythrophagocytosis along with hemorrhage and congestion (Fig. 3).

### Assessment of extracellular particles in secretions

Just prior to euthanasia, CVS and CVL were collected. Samples were collected with the aid of a vaginal speculum by either gently swabbing in the endocervical area with a sterile cottom swab or by lavaging the vaginal vault with a syringe filled with 3.0 mililiters of 1X PBS, respectively. The total yield of CVL fluid was recorded. Swabs were immediately placed in two mL of 1X Dulbecco’s PBS and within thirty minutes of collection were swirled in a circular fashion within the PBS and then repeatedly pressed against the side of their respective 15 mililiter conicals to ensure optimal liberation of secretions; lavage fluid was collected into a 15 mililiter conical. Normal CVL samples were pooled by species prior to analysis. Both types of samples were centrifuged at 1,000xg for 15 min to remove cells and large debris. Following this first spin, supernatant from each of the sample types was saved, diluted 1:10 with 1X phosphate buffered saline (PBS) and subjected to single particle tracking with a NanoSight NS-500^1^ system. At least five 20-s videos were recorded of each sample at a camera setting of 14, and files were analyzed at a detection threshold of ten using NanoSight software version 2.2.

Samples subsequently underwent high-speed ultracentrifugation (110,000xg for 70 min at 4 °C) using a Sorvall Discovery 100SE^2^ instrument and a Sorvall AH-650^2^ swinging-bucket rotor (k-factor =53). After carefully pouring off supernatant, pellets were resuspended in 500 ul PBS using an optimized resuspension protocol. Both sets of samples were diluted at a ratio of 1:10 as well and observed using the same camera and detection threshold settings as were used for the pre-ultracentrifugation observation (described above).

Particle counts of samples from the endometriosis case were compared with CVL and CVS data obtained from four other individuals (two rhesus macaques and two pigtailed macaques, *Macaca nemestrina*) that had no notable reproductive tract history or pathology at necropsy, using the same protocols. Histologic examination of endometrial tissue from the 4 reproductively normal animals revealed that the animals were either in the proliferative phase (*n* = 3) or early secretory (*n* = 1) phase of the menstrual cycle, allowing for the best physiologic comparison with respect to the presence of endometrial cells observed in endometriosis [28–30].

## Results

**Fig. 4:**
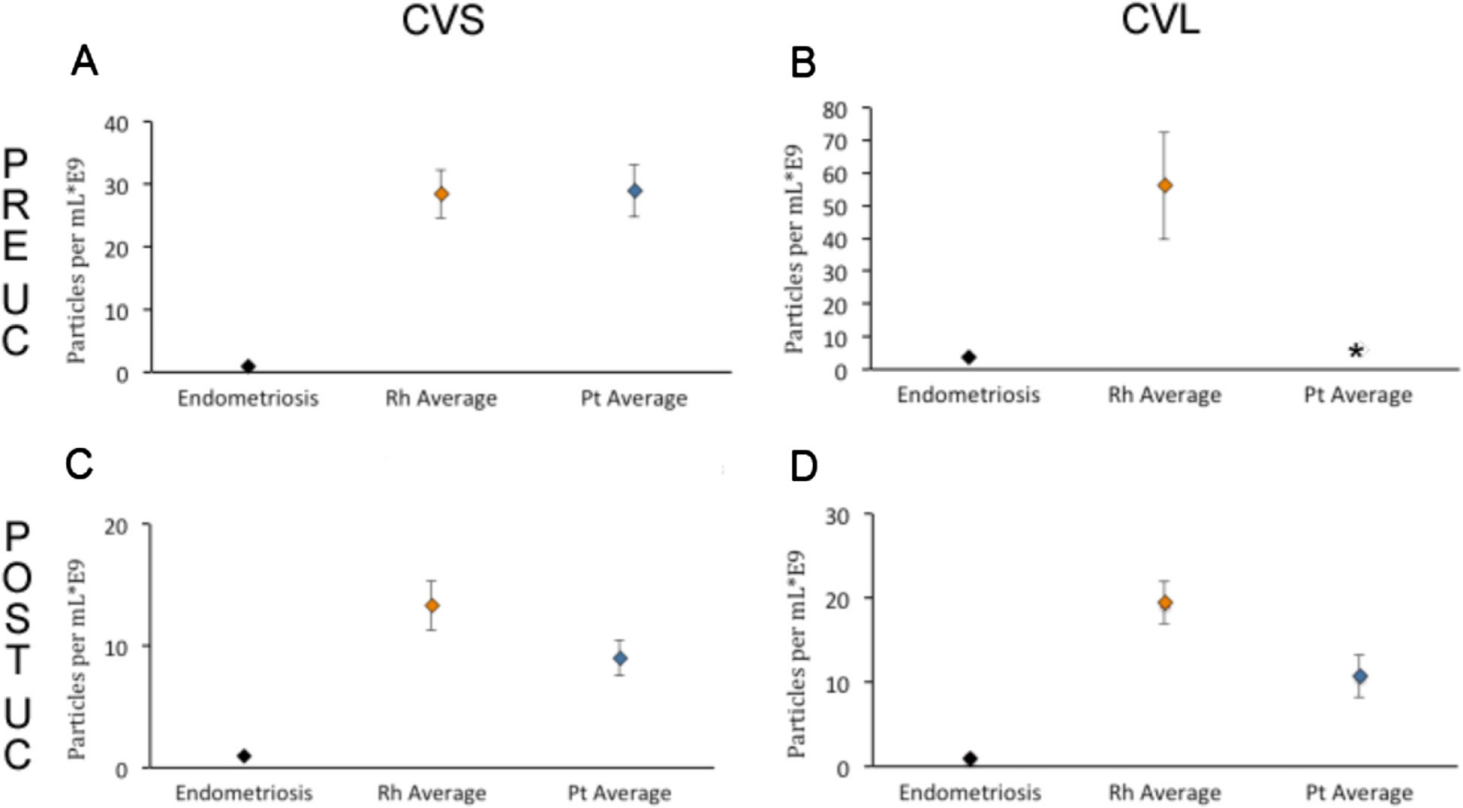
Pooled Data from Cervico-Vaginal Swabs and Cervico-Vaginal Lavage. NTA demonstrated a drop in particles per milliliter in pooled samples from two rhesus macaques (Rh) and two pig-tailed (Pt) macaques as compared to the number of particles observed in the animal with endometriosis. **a** and **b** compare CVS and CVL particles per milliliter at low-speed centrifugation (PRE UC) and **c** and **d** compare particles observed post-ultracentrifugation (POST UC). Asterisk in panel **b**: a software malfunction resulted in incomplete acquisition of data and loss of sample. Error bars for averages in CVS samples indicate standard deviation of readings across multiple animals and deviation between readings for the case endometriosis and CVL pooled samples

After removal of cells and large debris from the CVS fluid by low-speed centrifugation, on average approximately 30-fold fewer extracellular particles were found in the endometriosis sample than in samples from reproductively healthy rhesus and pigtailed macaques (Fig. 4a). In corresponding CVL samples, similar but less pronounced differences were obtained (Fig. 4b).

Stepped ultracentrifugation is the most widely used and accepted method for EV enrichment [31], although other particles may co-purify with EVs [31–35]. Following a standard ultracentrifugation protocol, the resulting EV-enriched particle population was approximately nine- to 13-fold more abundant in CVS samples from reproductively healthy animals than in the case of endometriosis (Fig. 4c), and approximately ten- to 20-fold more abundant in corresponding CVL (Fig. 4d).

## Conclusions

Here, we report nanoparticle tracking analysis of extracellular particles including EVs in cervicovaginal samples associated with a verified case of an ongoing endometrial disease process. In samples collected by both lavage and swab, particle concentration was markedly decreased in the case of endometriosis as compared with reproductively healthy animals.

In this case report, we are admittedly unable to address several important issues. First, since results were obtained after the disease had taken its full course, it is unclear whether particle counts would be altered in early stage disease. Carefully designed longitudinal studies of primate endometriosis models will be needed. Second, our studies focused on quantifying particles using the limited volume of sample we could obtain from the case, to the exclusion of other techniques that might have provided more information on the subtypes (“exosomes,” “microvesicles,” etc.), origins, and potential functions of particles. We chose NTA as one of the first and simplest readings that could be done, one that would thus be potentially useful in the clinic. However, many biological questions remain unanswered. EM and immunoaffinity assays, likely using pooled samples, will be needed to identify the various sources and types of particles found in the cervicovaginal compartment—sources, we would note, that might include the microbiome, not just the host. Third, we do not know what is causing the observed decrease. The mechanisms for altered EV and other particle release from the endometrium and the vaginal epithelium—in disease or during cycling or pregnancy—must be identified through longitudinal sampling (as mentioned above) of different compartments. For example, to ascertain the effects of normal or altered cycling, one might wish to examine particle populations in paired vaginal and intrauterine swabs along with hormone levels to [36]. Finally, we have not yet profiled cervicovaginal particles for amplifiable biomarkers, such as RNA molecules.

Although much more remains to be learned about the nature and functions of the particles and vesicles we found, our findings suggest that particulates in the cervicovaginal compartment may provide novel, minimally invasive diagnostic tools for reproductive tract disease. We trust that these results will spur larger studies in animal models of endometriosis as well as in human cohorts.

## Abbreviations

CVS: Cervicovaginal Swab
CVL: Cervicovaginal Lavage
PBS: Phosphate Buffered Saline
BCS: Body condition score

## Footnotes

1 Malvern, Worcestershire, United Kingdom

2 ThermoFisher, Waltham, Massachusetts, USA
